# Simultaneous 3D left and right coronary artery vessel wall imaging

**DOI:** 10.1186/1532-429X-13-S1-P239

**Published:** 2011-02-02

**Authors:** Andrew D Scott, Jennifer Keegan, David N Firmin

**Affiliations:** 1 Imperial College London, London, UK; 2 The Royal Brompton and Harefield NHS Foundation Trust, London, UK

## Objective

To perform 3D imaging of the left and right coronary vessel walls in a single interleaved acquisition.

## Background

Vessel wall imaging is commonly gated to alternate R-waves, in order to increase SNR and minimise artefacts due to RR-interval variation. In previous work with 2D carotid artery wall imaging, the resulting redundant RR-interval has been used to acquire additional parallel slices[1]. We postulate that the redundant RR-interval can be used to image an additional volume in 3D coronary artery wall imaging. In conjunction with highly efficient beat-to-beat respiratory motion correction (B2B-RMC)[2], this could allow high resolution 3D acquisitions of both the left and right coronary walls in ~10minutes.

## Methods

**Figure 1:**
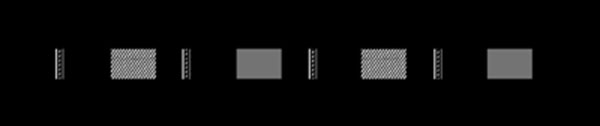
Schematic diagram of the simultaneous left and right 3D coronary vessel wall imaging sequence. In each cardiac cycle, the dark blood preparation non-selectively inverts all tissue and selectively reinverts tissue slabs covering the left and right coronary arteries. Left and right coronary artery wall data are acquired on odd and even cardiac cycles respectively. Each imaging block includes the acquisition of a low resolution 3D fat-excitation dataset which is used to track respiratory motion for B2B-RMC [2].

A free-breathing 3D spiral coronary artery wall imaging sequence was modified in order to acquire two high resolution 3D volumes (0.7x0.7x3mm resolution, 8 slices (reconstructed to 16x1.5mm), acquisition duration 600 cardiac cycles assuming 100% respiratory efficiency). The first and second volumes were imaged on odd and even cardiac cycles respectively (figure 1). The slab selective dark blood preparation was modified to reinvert two slabs every cardiac cycle. Respiratory motion compensation was performed independently for each artery using retrospective B2B-RMC which has a respiratory efficiency of nearly 100% [2],[3].

Acquisitions were performed in healthy subjects using a Siemens Avanto 1.5T scanner. The first and second volumes were positioned to obtain cross-sectional left and right coronary images respectively. The first reinversion slab was positioned to selectively reinvert the left coronary artery whilst avoiding reinversion of aortic blood and minimising reinversion of the tissue imaged in the right coronary artery volume. The second reinversion slab was similarly positioned for the right coronary artery. Inversion time was 400ms which is optimal for single R-wave gating at 60beats/minute.

## Results

**Figure 2:**
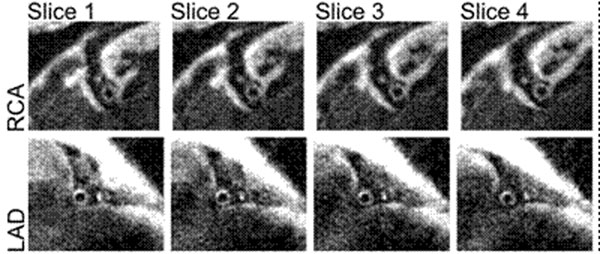
Four contiguous slices from the right (top) and left (bottom) coronary artery volumes acquired simultaneously in 629 cardiac cycles using a dark blood 3D spiral acquisition with B2B-RMC. The respiratory efficiency was 95%.

Left and right coronary artery wall images obtained simultaneously in 629 cardiac cycles are shown in figure 2. The respiratory efficiency was 95%. Image quality, respiratory motion compensation and blood suppression is good in both arteries.

## Conclusions

We have demonstrated simultaneous left and right coronary artery wall imaging in the duration required to image a single artery. Used in conjunction with B2B-RMC, this has permitted high resolution 3D imaging of both the left and right coronary walls in approximately 10minutes. Future work will include a comparison with conventional alternate R-wave gating acquisitions and an investigation of the robustness of the technique to changes in RR interval.

## References

[B1] Parker MRM 2002

[B2] Keegan JMRI 2007

[B3] Scott JMRI in press

